# Long-term musculoskeletal function after Open Pelvic ring fractures in Children (OPEC); a multicentre, retrospective case series with follow-up measurement

**DOI:** 10.1016/j.tcr.2024.101050

**Published:** 2024-06-05

**Authors:** A.H.M. Mennen, E.M.M. Van Lieshout, P.A. Bisoen, F.W. Bloemers, A.E. Geerlings, D. Koole, M.H.J. Verhofstad, J.J. Visser, D. Van Embden, M.G. Van Vledder

**Affiliations:** a Erasmus MC, University Medical Center Rotterdam, Trauma Research Unit Department of Surgery, dr. Molewaterplein 40, Rotterdam, the Netherlands; b Amsterdam UMC location University of Amsterdam, Department of Surgery, Meibergdreef 9, Amsterdam, the Netherlands; c Amsterdam UMC location Vrije Universiteit Amsterdam, Department of Surgery, De Boelelaan 1117, Amsterdam, the Netherlands; d Erasmus MC, University Medical Center Rotterdam, Department of Radiology, dr. Molewaterplein 40, Rotterdam, the Netherlands

**Keywords:** Open Pelvic fracture, Children, Long-term outcomes, Musculoskeletal dysfunction, Neurological dysfunction, Urogenital dysfunction

## Abstract

**Background:**

The proportion of Open Pelvic fractures in the paediatric population is relatively high. While operative fixation is the primary approach for managing Open Pelvic fractures in adults, there is limited literature on treatment outcomes in Children, particularly regarding long-term musculoskeletal, neurological, and urogenital function.

**Methods:**

This multicentre case series included paediatric patients (<18 years old) with Open Pelvic ring fractures treated at one of two major trauma centres in the Netherlands between January 1, 2001 and December 31, 2021. Data collection involved clinical records and long-term assessments, including musculoskeletal function, growth disorders, urogenital function, sexual dysfunction, and sensory motor function.

**Results:**

A total of 11 patients were included, primarily females (73 %), with a median age at trauma of 12 years (P_25_–P_75_ 7–14). Most patients had unstable Pelvic ring fractures resulting from high-energy trauma. Surgical interventions were common, with external fixation as the main initial surgical approach (*n* = 7, 70 %). Complications were observed in eight (73 %) patients. Musculoskeletal function revealed a range of issues in the lower extremity, daily activities, and mental and emotional domain. Long-term radiologic follow-up showed high rates of Pelvic malunion (*n* = 7, 64 %). Neurological function assessment showed motor and sensory function impairment in a subset of patients. Urogenital function was moderately affected, and sexual dysfunction was limited with most respondents reporting no issues.

**Conclusion:**

Paediatric Open Pelvic fractures are challenging injuries associated with significant short-term complications and long-term musculoskeletal and urogenital issues. Further research is needed to develop tailored treatment strategies and improve outcomes of these patients.

## Introduction

Pelvic ring fractures in Children, although relatively uncommon, pose significant challenges due to their potential for severe morbidity and mortality. These fractures are typically the result of high-energy trauma and account for approximately 0.5 % to 7.0 % of all paediatric fractures [1,2]. Mortality rates associated with these fractures vary widely in the literature, ranging from 1.4 % to 25 %, with an average rate of 6.4 % [1,2]. In addition to the risk of mortality, paediatric patients with Pelvic ring fractures may experience long-term complications, such as musculoskeletal dysfunction, growth deformities, and neurological and urogenital impairments [[1], [2], [3]].

Whereas Open Pelvic fractures are rare in adults (2–4 %), they account for a higher proportion of Pelvic ring fractures in the paediatric population, reaching up to 12.9 % [1,2]. An Open Pelvic fracture is characterized by a direct communication between the fracture and the external environment, often occurring through the rectum, genitals, or skin. In comparison to closed fractures, Open Pelvic fractures carry a higher risk of urogenital complications, including bladder control problems and sexual issues.

Historically surgeons mostly treated Open fractures with external fixation only, since the general idea was that more invasive surgical procedures would further contaminate the area [4,5]. However, several studies have shown that early fixation is essential, since any major motion or shearing forces will continue to disrupt the soft tissues and prevent definitive soft tissue healing [6,7]. Furthermore, a mechanically unstable Open Pelvic fracture is associated with a ten-fold increased risk of infection due to the large hematoma associated with the unstable fracture [8]. The current management of Open Pelvic fractures in all patients is very much dependent on the extend and type of soft tissue and concomitant injuries e.g. urogenital, and mostly consist of early surgical fixation of the Pelvic fracture and soft tissue injuries.

For Children, however, the available literature on the treatment and short-term clinical outcomes of paediatric Open Pelvic fractures is scarce, and limited to a few case reports [[9], [10], [11]]. Moreover, there is a lack of data concerning long-term musculoskeletal function, growth deformities, and neurological and urogenital function following (non)operative treatment of Open Pelvic ring fractures in Children.

Therefore, the aim of this study was to assess long-term patient-reported musculoskeletal function in patients who sustained an Open Pelvic ring fracture when they were aged <18 years. Secondary aims included the assessment of growth disorders, urogenital function, sexual dysfunction, and sensomotoric function of the lower extremities after clinical treatment.

## Methods

### Study design and study setting

This multicentre case series investigated paediatric patients who sustained a Pelvic ring fracture at ≤18 years of age and were treated between January 1, 2001 and December 312,021. All relevant patients were identified by searching the hospital's electronic patient files of both hospitals, and the Dutch national trauma registry.

Patients were eligible for inclusion if the age at trauma was below 18 years, if they had sustained an Open Pelvic ring fracture, and when written informed consent for this study was given by the patient and/or parents. Exclusion criteria were <30 days of follow-up post-trauma available in the patient's file, unknown contact details for patient (and/or parents), and insufficient comprehension of Dutch language to understand the questionnaires or other study information.

### Outcome measures and data collection

The primary outcome measure of this study was the Short Musculoskeletal Functional Assessment (SMFA). Secondary outcome parameters were the presence of growth disorders, urogenital function (VSSDES), sexual dysfunction (IIEF-5 in males and FSFI in females), motor function of the lower extremities (MRC scale,) and sensory function of the lower extremities (soft touch and pin prick test for gnostic and vital sensitivity, respectively).

Data consisted of retrospective collection of clinical data and a single measurement of long-term musculoskeletal function during a follow-up appointment. Patients were invited for a single visit to the outpatient department. During this visit, a Pelvic radiograph was made to assess growth disorders, and sensomotoric loss was assessed using a non-invasive soft touch and pin prick test for gnostic and vital sensitivity, respectively. In addition, patients were asked to complete three questionnaires; Short Musculoskeletal Functional Assessment (SMFA) for musculoskeletal function, Vancouver Symptom Score for Dysfunctional Elimination Syndrome (VSSDES) for urinary incontinence, and the International Index of Erectile Function (IIEF-5) in males or Female Sexual Function Index (FSFI) in females for sexual dysfunction. Other study outcomes that were collected from the electronic patient files were patient characteristics, trauma and injury characteristics, details of the index treatment, post-operative details, and details at follow-up.

### Definitions

Motor function of the lower extremities was assessed using manual muscle testing and was graded according to the Medical Research Council (MRC) scale [12]. Motor function of the sciatic nerve (flexion), femoral nerve (extension), obturator nerve (adduction), and the gluteus medius (abduction) were graded as MRC 0–5 for both the left and right leg. A MRC score of 5 is normal, 4 is strong, and 0–3 poor. Sensory function of the lower extremity was assessed using soft touch and pin prick test. Both gnostic (soft touch) and vital (pin prick) sensibility of the sciatic nerve, femoral nerve, and obturator nerve were scored for the left and right leg separately.

The Short Musculoskeletal Functional Assessment (SMFA) consists of 46 items. The Dutch version of the SMFA (SMFA-NL) can be divided into four subscales: the upper extremity dysfunction (6 items), lower extremity dysfunction (12 items), problems with daily activities (20 items) and mental and emotional problem (8 item) subscales [13]. All items were scored on a 5-item Likert scale, ranging from 1 (good function/not bothered) to 5 (poor function/extremely bothered). The SMFA score was calculated for each subscale using the formula: ((sum of all items/number of items)-1)*maximum score. The overall score as well as the subscale scores range from 0 to 100 points. Higher scores refer to greater disability [13,14].

Urogenital function was assessed by the Vancouver Symptom Score for Dysfunctional Elimination (VSSDES) [15]. This is a 14-item measure of which the last item, which evaluates the difficulty of the measure, is not included in the score. All remaining items were weighted equally and responses were scored using a 5-point Likert scale. Scores ranged from 0 (no complaints) to 4 (severe symptoms), with total scores ranging from 0 to 52. Higher scores indicate more severe symptoms. This study used a validated Dutch version of this score [16].

The International Index of Erectile Function (IIEF-5) and Female Sexual Function Index (FSFI) were used to assess sexual dysfunction in males and females, respectively [17,18]. In the IIEF-5 a score of 0–5 is awarded to each of the 5 items. The overall score is a summation of the 5 items and ranges from 0 to 25. A score of 1–7 represents severe ED, 8–11 moderate ED, 12–16 mild-moderate ED, 17–21 mild ED, and 22–25 no ED. This study used a validated Dutch version of this score [19]. The FSFI is a 19-item question about sexual functioning in women. The items represent five domains and each item has 5 or 6 answer options. Total scores range from 2 to 36, with a higher score indicating a better sexual function index. A score of 26 or less indicates risk for sexual dysfunction. This study used a validated Dutch version of this score [20].

The Faringer classification was used to describe the localisation of the soft-tissue injuries [21]. The Gustilo-Anderson and the Jones-Powell classification were used to describe the severity (and localisation) of the soft-tissue injury [22,23]. The Gustilo-Anderson classification was initially developed for Open fractures of long bones, so it should be noted that this does not fully translate to Open Pelvic fractures due to the differences in the nature of these injuries. In this study any soft-tissue injury directly related to the Open Pelvic fracture which required surgical intervention was classified as a Gustilo-Anderson type 3a. Fracture patterns were classified using the Tile classification and the Torode & Zieg classification [24,25].

### Data analysis

No formal sample size calculation was made. All patients meeting the eligibility criteria were invited to participate. Given the low prevalence of Open Pelvic ring fractures, no >10 paediatric patients with Open Pelvic ring fracture were expected per site. At this sample size and with the expected heterogeneity in age and injuries across the participants, the study was explorative rather than hypothesis testing. Data were analysed using the Statistical Package for the Social Sciences (SPSS) version 28.0 (SPSS, Chicago, Ill., USA). Normality of continuous data was tested with the Shapiro-Wilk test. Missing values were not imputed. Descriptive analysis was performed for the entire group. The mean and SD (parametric data) or the median and percentiles (non-parametric data) were reported for the combined score as well as the SMFA subdomains. In order to compare the SMFA subdomain scores with the population norms, a one-sample *t*-test (parametric) or a one-sample Wilcoxon Signed Rank test (non-parametric) was performed. Given the expected low sample size, heterogeneity, and the explorative nature of this study, no further statistical analysis was done.

## Results

In total 16 patients were identified as eligible for inclusion. One patient could not be reached due to out of date contact information, and four patients declined participation. This led to an inclusion of 11 patients in total.

### Patient and treatment characteristics

Table 1 shows details on patient and injury characteristics. The majority of the patients was female (*n* = 8, 73 %) and the median age at trauma was 12 years (P_25_–P_75_ 7–14). The most common mechanism of trauma was bicycle versus truck or bus. None of the patients had any prior medical history that could interfere with their functional independence. Most patients (*n* = 10, 91 %) had a T&Z type 4 unstable Pelvic ring fracture. One patient had an isolated Open iliac wing fracture. According to the Tile classification, type B3 (e.g. bilateral rotational instability) was the most common fracture pattern (*n* = 6, 55 %). The majority of the soft tissue injuries were located in Faringer zone 1 (*n* = 7, 64 %). Most patients had a Gustilo-Anderson type 3a injury (*n* = 9, 82 %) and Jones-Powel type 3 (n = 6, 55 %). Rectal injuries and anal sphincter injuries were seen in 5 (45 %) and 4 (36 %) patients, respectively. Vaginal lacerations were seen in 5 (45 %) patients, urethra injuries in 4 (36 %), and bladder injuries in 1 patient (9 %). In 3 (27 %) patients there was an avulsion of the Pelvic floor muscles and 2 (18 %) patients had extensive skin defect (e.g. deglovements). Nerve injuries in the lower legs were seen in 3 (27 %) patients; two (18 %) patients had a severe lumbosacral plexus injury with sensory and motor function loss, and one (9 %) patient sustained a partial ischiadic nerve injury. There were two (18 %) patients with concomitant acetabular fractures. The mean ISS was 33 (SD 12.2). All patients (*n* = 11, 100 %) had concomitant injuries and fractures.

**Table 1:** Details of the patient and injury characteristics of the individual paediatric patients with an Open Pelvic ring fracture.

	Sex	Age	Year TRM	MOI	Tile	T&Z	FAR	G-A	J-P	Soft-tissue injuries	Nerve injuries	ISS
1	F	12	2020	Bike vs. truck or bus	B3	4	1	4	3	Pelvic floor muscles, rectum, anal sphincter	LS plexus injury (L5 + S1) left	48
2	M	14	2004	Bike vs. truck or bus	B3	4	2	4	2	Bladder	LS plexus injury (L3) right	26
3	F	12	2002	Fall from height	B3	4	3	4	2	Vagina		34
4	F	9	2010	Bike vs. truck or bus	B3	4	1	4	3	Rectum, anal sphincter, vagina		18
5	M	18	2019	Train-related accident	A2	2	3	1	1	Skin (>10 cm)		41
6	F	12	2019	Bike vs. truck or bus	C2	4	1	4	3	Rectum, anal sphincter, urethra, vagina	N. ischiadicus left	33
7	F	4	2020	Bike vs. truck or bus	B2	4	1	4	3	Pelvic floor muscles, rectum, urethra		41
8	F	14	2020	Fall from horse	B3	4	1	4	2	Vagina		10
9	F	7	2006	Pedestrian vs. truc or bus	B3	4	1	4	3	Rectum, anal sphincter, urethra, vagina		50
10	F	12	2014	Bike vs. truck or bus	B2	4	3	2	2	Skin (>10 cm)		34
11	M	6	2015	Bike vs. truck or bus	B2	4	1	4	3	Pelvic floor muscles + urethra		26

FAR, Faringer classification; G-A, Gustilo Anderson classification; ISS, Injury Severity Score; J-P, Jones Powell classification; LS, Lumbosacral; MOI, mechanism of injury; T&Z, Torode & Zieg classification; TRM, trauma.

A Pelvic circumferential compression device was used in 10 (91 %) patients. In two patients there was no information about receiving a blood transfusion. Nine patients received a median of 4 (IQR 2–10) packed cells, three patients received a median of 1 (IQR 1–1) platelet transfusion, and seven patients received a median of 4 (IQR 2–8) platelet transfusion.

Details on of the surgical treatment and soft-tissue repair is shown in Table 2. Ten (91 %) patients underwent fracture fixation; seven (70 %) were initially treated with external fixation and two (20 %) with immediate Open reduction and internal fixation. Four (57 %) of the patients who were initially treated with external fixation eventually underwent Open reduction and internal fixation as definitive fixation method, and one (14 %) patient underwent a hemipelvectomy.

**Table 2:** Details of type of fracture and soft-tissue surgery, complications <30 days, and re-operations <30 days and >30 days of the individual paediatric patients with an Open Pelvic ring fracture.

	Treatment	Details ORIF	Surgery soft-tissue injuries	Complications	*Re*-operation < 30 days	Re-operation > 30 days
1	EXFIX, then ORIF	Anterior plate + posterior 2× SI-screw	Colostomy and repair perineum, anal sphincter	SIRS, hypovolemic shock, ileus, infected OSM	Multiple VAC dressings + wound nettoyage + fixation humerus Fx	Multiple VAC changes + wound nettoyage + reversal colostomy + removal SI-screw + SSG upper leg
2	EXFIX, then ORIF	Posterior 1× SI-screw	Repair bladder rupture	Compartment syndrome		
3	EXFIX, then ORIF	Posterior 2× SI-screw	Wound debridement right Pelvic crista			
4	EXFIX		Colostomy and repair vagina, rectum, anal sphincter, perineum	UTI		Reversal colostomy
5	ORIF	Anterior plate	Wound debridement left Pelvic crista	Pulmonary embolism		
6	EXFIX, then ORIF	Anterior plate + posterior 2× plate over SI-joint +1× SI-screw	Colostomy and ALT free flap for soft-tissue coverage	Abces	Abces drainage	Abces drainage + revision and reversal of colostomy
7	ORIF	Posterior 1× plate ilium +2× SI-screw	Repair rectum, vagina, perineum			
8	ORIF	Anterior plate + posterior 2× SI-screw	Repair vagina	Vaginitis, wound infection		Removal of OSM
9	EXFIX, then hemipelvectomy		Colostomy			Vaginal reconstruction
10	Conservative		Colostomy	Wound infection		Reversal colostomy
11	EXFIX		Colostomy and repair urethra, perineum			Reversal colostomy

ALT, anterolateral thigh; EXFIX, external fixation; Fx, fracture; ORIF, Open reduction internal fixation; OSM, osteosynthesis material; SSG, split skin graft; SIRS, systemic inflammatory response syndrome; UTI, urinary tract infection; VAC, vacuum assisted closure.

Of the patients who underwent surgical stabilization of their Pelvic fracture, 9 (90 %) were operated on the same day as they arrived at the hospital. The two patients who were operated on day 1 and day 3 post-trauma underwent immediate Open reduction internal fixation.

All patients (100 %) had surgical repair of their soft-tissue injuries. Most patients had their surgical repair direct or within 1 day after the trauma (*n* = 7, 70 %). The median time to weight bearing was 1,6 months (P_25_–P_75_ 0,72–2,3).

One patient was directly admitted to the surgical wards, all other patients were initially admitted to the ICU (*n* = 10, 91 %). The median stay at the ICU was 9.5 days (P_25_–P_75_ 2_−_31 days). One patient had to be readmitted to the ICU for a total of one day during their hospital admission.

Details on complications and reoperations are shown in Table 2. Eight patients (73 %) had a complication <30 days of admission. Four (36 %) patients had to undergo a re-operation <30 days, and 7 (64 %) patients had to undergo a re-operation >30 days.

The median length of hospital stay was 26 days (P_25_–P_75_ 16–60). After discharge seven (64 %) patients were admitted to a physical rehabilitation centre and four (36 %) directly returned home. The median follow-up time is 23 months (P_25_–P_75_ 11–55), however it should be noted that in four of the 11 patients (36 %) follow-up has not been completed.

### Description and imaging of two cases

**Fig. 1.**
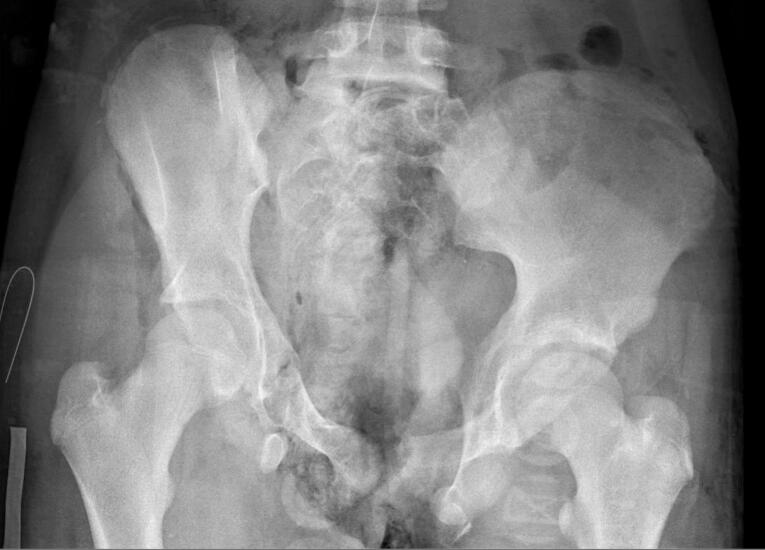
Anteroposterior Pelvic radiograph of 12 year old female with a Tile type C2 Pelvic fracture.

**Fig. 2.**
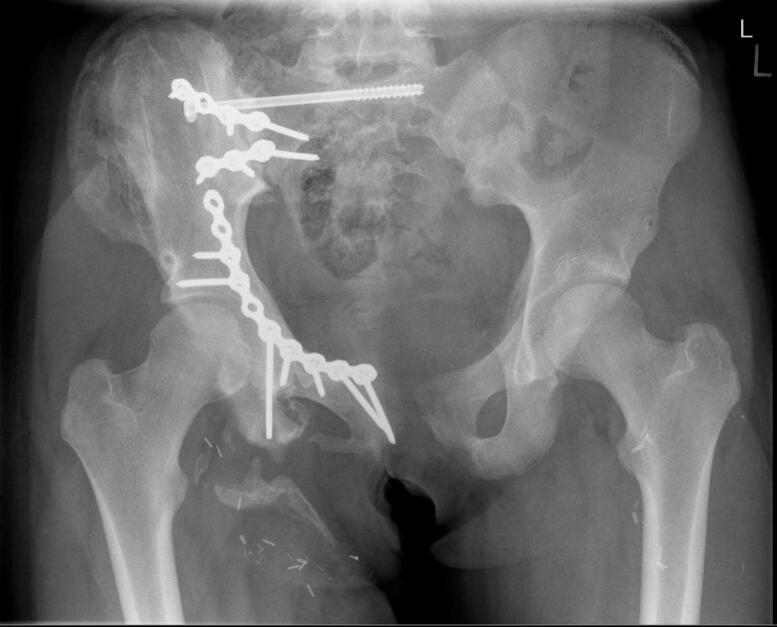
Anteroposterior Pelvic radiograph 6 weeks after Open reduction and internal fixation.

Case 1: a 12 year old female patient who sustained an Open Pelvic fracture (Tile type C2) with soft tissue injuries to the Pelvic floor muscles, anal sphincter, urethra, and vagina after being hit by a truck while driving her bike. Fig. 1 shows an anteroposterior Pelvic radiograph of the Tile type C2 Pelvic fracture, Fig. 2 shows an anteroposterior Pelvic radiograph of the Pelvic fracture 6 weeks after Open reduction and internal fixation by sacroiliac screw and plate osteosynthesis. The loose bone fragment of the ischial tuberosity was resected due to pain complaints two years after the initial surgical fixation.

**Fig. 3.**
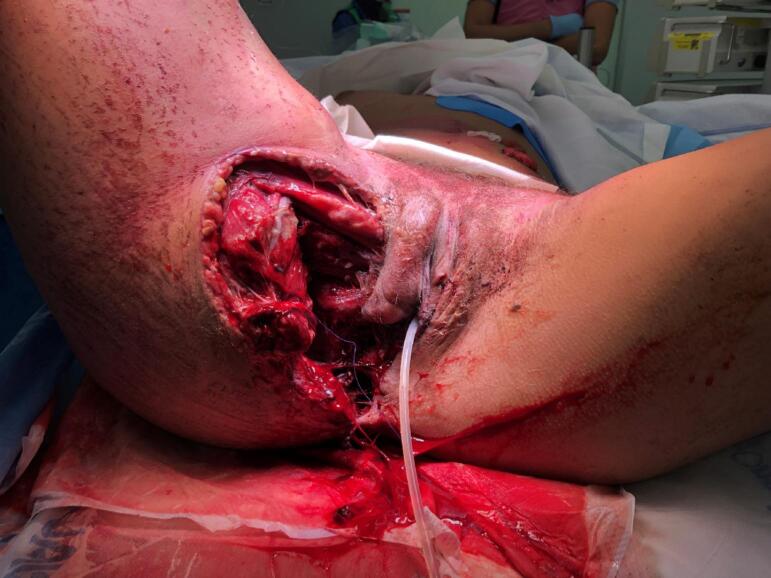
Soft-tissue injuries before reconstruction.

**Fig. 4.**
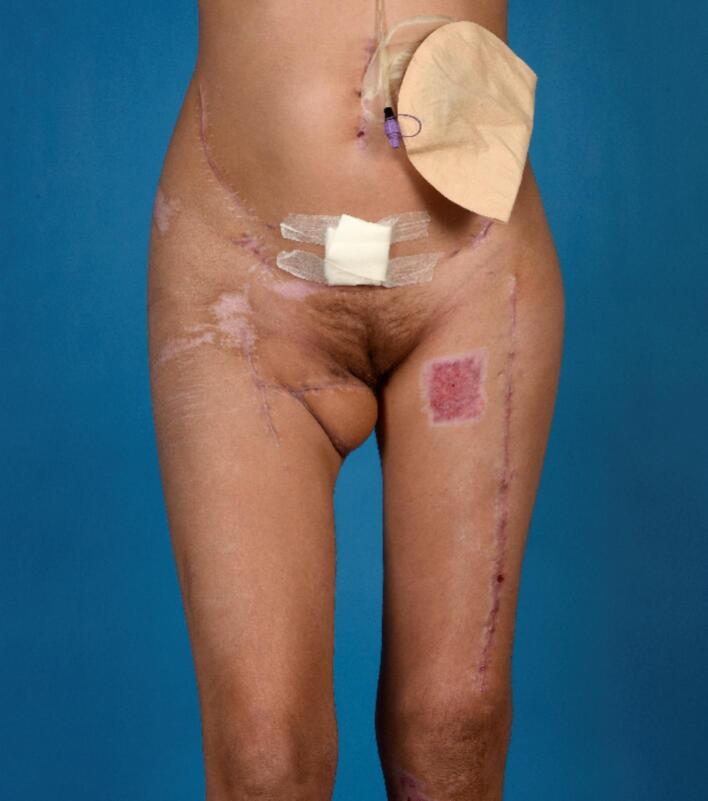
Soft-tissue status 3 months after the trauma and reconstruction.

Fig. 3 shows the soft-tissue injuries before reconstruction, and Fig. 4 shows the soft-tissue status 3 months after the trauma and reconstruction.

**Fig. 5.**
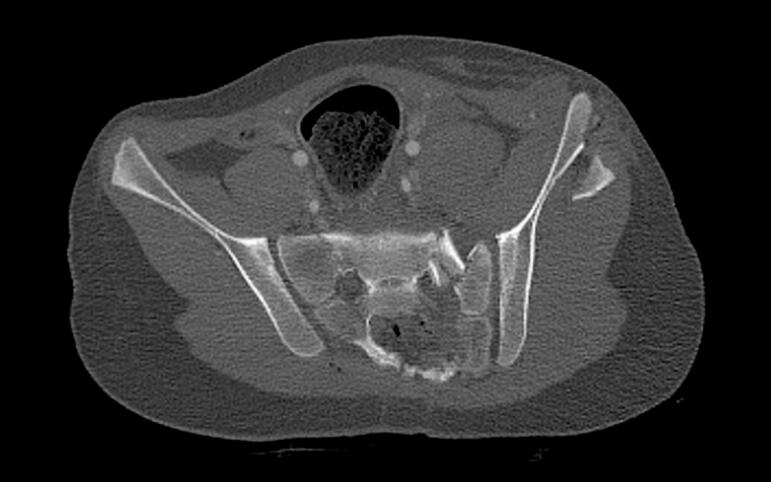
Transversal view of CT-scan of a 12 year old female who sustained a Tile type B3 Pelvic fracture.

**Fig. 6.**
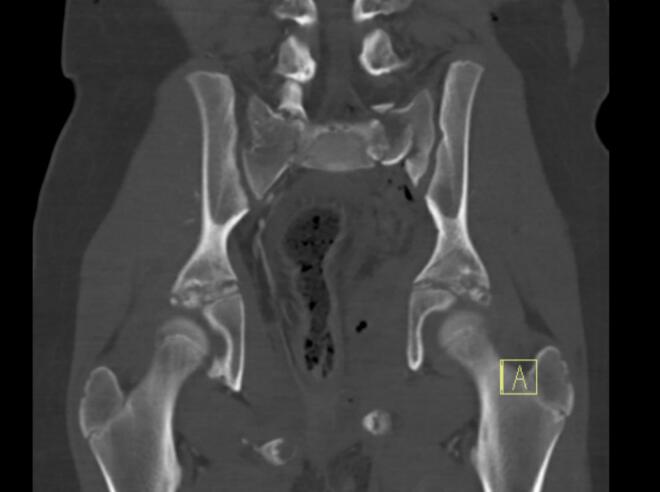
Coronal view of CT-scan of a 12 year old female who sustained a Tile type B3 Pelvic fracture.

**Fig. 7.**
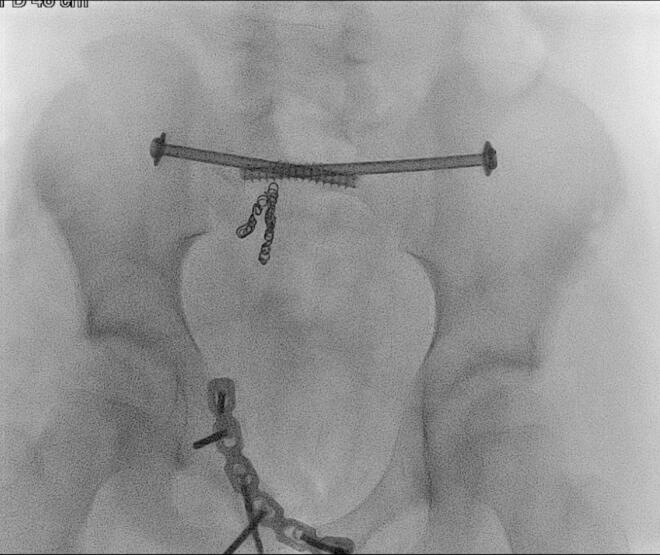
Per-operative inlet Pelvic radiograph during Open reduction and internal fixation.

**Fig. 8.**
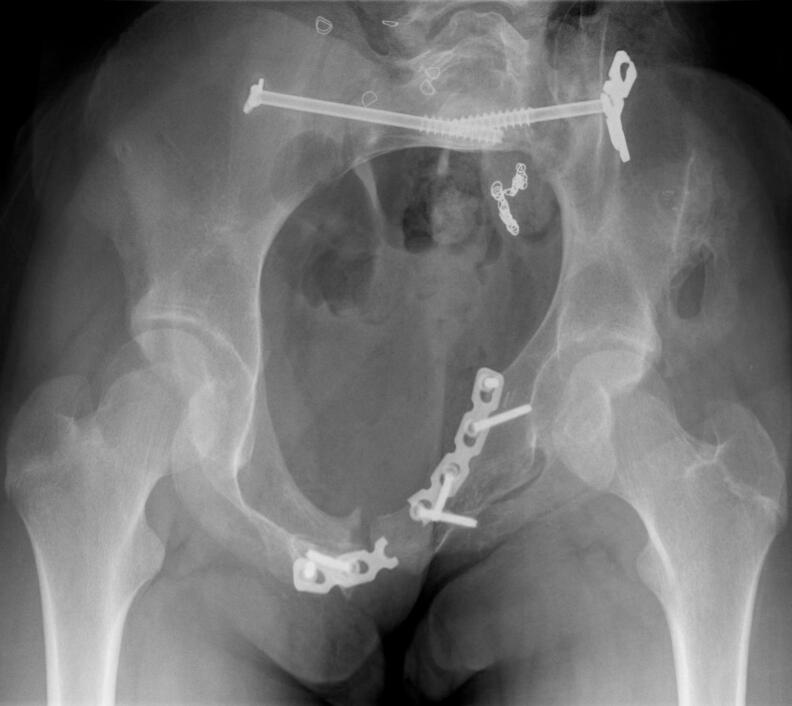
Inlet Pelvic radiograph 2.5 years after surgical fixation.

Case 2: a 12 year old female who sustained an Open Pelvic fracture (Tile type B3) with soft tissue injuries to the Pelvic floor muscles, rectum, and anal sphincter, after being hit by a bus while driving her bike. Fig. 5 shows the transversal view and Fig. 6 the coronal view of the computed tomography scan (CT-scan) showing the Tile type B3 Pelvic fracture. Fig. 7 shows an per-operative inlet Pelvic radiograph during Open reduction and internal fixation by bilateral sacroiliac screw and anterior plate osteosynthesis with adequate reposition. Fig. 8 shows the inlet Pelvic radiograph 2.5 years after surgical fixation. The anterior plate broke <5 months post-operative but was not removed.

### Long-term musculoskeletal, neurological, and urogenital function

All included patients visited the outpatient department to assess potential loss of function, got a X-ray of the pelvis (unless a recent Pelvic X-ray was available), and completed the questionnaires. The visit was on average 95 months after their trauma (range 17–247 months).

### Motor and sensory function of the lower extremities

The motor function of the ischiadic, femoral, obturator, and gluteal nerve, measured using the MRC scale, was normal in five (45 %) patients. One patient had decreased motor function of the left ischiadic nerve, but was able to move against gravity and light resistance. One patient had decreased motor function of the right ischiadic and femoral nerve, but was able to move against gravity and light resistance. One patient had decreased motor function of the left ischiadic and femoral nerve and right ischiadic nerve, but was able to move against gravity and light resistance. One patient had decreased motor function of the right obturator and gluteal nerve, but was able to move against gravity and light resistance. One patient had decreased motor function of all nerves in the left leg, but was able to move against gravity and light resistance. One patient had poor function of all nerves in the left leg and the motor function of the right leg could not be scored since it was amputated.

Evaluating the neurological function, 8 patients (73 %) have normal gnostic and vital sensibility of the ischiadic, femoral, obturator, and gluteal nerve in both legs. One patient had decreased gnostic and vital sensibility of the ischiadic nerve of the left leg. One patient had decreased gnostic and vital sensibility of the femoral and obturator nerve of the right leg. One patient had normal neurological function of the left leg, but the right leg could not be scored due to amputation.

### Radiological findings

Two patients (18 %) had a growth disorder on their Pelvic X-ray; one patient with an underdeveloped right hemipelvis, and one patient with a hypoplastic pubic bone on the right. Seven patients (64 %) had malunion of their pelvis fracture; posterior Pelvic asymmetry and rotational deviation was seen in four patients, and symphysis pubis deformity was also seen in four patients. Three patients (27 %) had non-union of loos bone fragments situated in different areas of the pelvis; one patient with non-union of a major bone fragment of the iliac wing, and two patients with non-union of the superior or inferior pubic ramus. Symphysis pubis joint ankylosing was seen in 2 patients (18 %) and of the SI-joint in 1 patient (9 %). Osteophytes of different Pelvic bones were seen in 5 patients (45 %). In one patient the osteosynthesis plate spanning over the symphysis has broken.

### Short Musculoskeletal Function Assessment (SMFA)

Table 3 shows the results of the SMFA questionnaire. All 11 patients returned the SMFA questionnaire. There were two items with a high number of missing values (*n* = 4, 36 %). These questions were about driving a car and sexual activities. The mean total SMFA score was 16 (SD 10.4). An analysis on the subscales of the Dutch SMFA was also performed to distinguish in which domains the dysfunctions were apparent. The mean score in the Lower Extremity Dysfunction domain was 14.4 (SD 10.7), in the Upper Extremity Dysfunction domain 0 (SD 0), the Problems with Daily Activities domain 17.9 (SD 14.8), and the Mental and Emotional problems domain 25.9 (SD 12.4).

**Table 3:** Details of musculoskeletal, urogenital, and sexual dysfunction scores of the individual paediatric patients with an Open Pelvic ring fracture.

	SMFA total	SFMA LE	SMFA UE	SMFA DA	SMFA ME	VSSDES	IIEF-5	FSFI
1	24.6	27.8	0.0	31.3	21.9	7,0		2,4
2	17.1	22.7	0.0	14.5	28.1	3,0	25,0	
3	7.8	6.8	0.0	2.6	28.1	19,0		31,5
4	14.7	4.6	0.0	16.2	37.5	9,0		31,7
5	34.1	27.3	0.0	42.1	50.0	6,0	23,0	
6	8.4	2.3	0.0	7.9	25.0	11,0		n/a
7	9.8	12.5	0.0	11.3	9.4	10,0		n/a
8	3.3	0.0	0.0	1.3	15.6	2,0		n/a
9	19.0	18.8	0.0	23.8	21.9	4,0		3,4
10	31.5	27.1	0.0	41.3	37.5	9,0		18,2
11	6.0	8.3	0.0	5.0	9.4	3,0	n/a	

DA, daily activities; FSFI, Female Sexual Function Index; IIEF-5, International Index of Erectile Function; LE, lower extremity; ME, mental and emotional; n/a, not available; SMFA, Short Musculoskeletal Functional Assessment; UE, upper extremity; VSSDES, Vancouver Symptom Score for Dysfunctional Elimination Syndrome.

### Urogenital function

The Vancouver symptom score for dysfunction elimination (VSSDES) was used to assess bladder and bowel symptoms. Table 3 shows the results of the VSSDES questionnaire. The total scores range from 0 to 52 with higher scores indicating more problems. The mean VSSDES score was 7,5 (SD 4.9).

### Sexual dysfunction

Table 3 shows the results of the Index of Erectile Function (IIEF-5) questionnaire and Female Sexual Function Index (FSFI) questionnaire. One male and two females did not complete the questionnaire since they were not sexually active. Both men who completed the IIEF-5 scored over 22 points, indicating that they had no erectile dysfunction complaints. Five (71 %) women completed the FSFI questionnaire. The mean FSFI score was 17.4 (SD 14.4). Two of the five women (40 %) who completed the questionnaire were not sexually active in the past month so scored overall low on the FSFI (mean 2.9, SD 0.7). The mean FSFI of the other three women was 27.1 (SD 7.7). Only one woman had a score of 26 or less indicating risk for sexual dysfunction.

## Discussion

This study investigated a series of 11 Children who suffered an Open Pelvic fracture, focusing on their clinical treatment within the first 30 days and assessing long-term musculoskeletal, neurological, and urogenital function outcomes. Our findings show a cohort of primarily female adolescents who suffered severe Pelvic injuries often resulting from traffic accidents involving large vehicles. The injuries were characterized by a high rate of soft tissue damage, nerve injuries, and concomitant injuries, with a substantial proportion of patients requiring surgical intervention, including Pelvic stabilization and soft tissue repair. The study population had a notable length of ICU stays and high complication rate, and the long-term follow-up showed a varied spectrum of outcomes. While many patients exhibited normal motor and sensory function of the lower extremities, some faced neurological deficits, growth disorders, and Pelvic malunion. Additionally, the assessment of urogenital and sexual function highlights the importance of addressing these aspects of recovery in paediatric patients with Open Pelvic ring fractures. The study emphasizes the need for comprehensive, long-term care and follow-up to optimize their functional outcomes and quality of life.

In general, Pelvic fractures in Children rarely cause significant bleeding and only 11 % to 39.5 % need a blood transfusion [26]. In contrast, almost all patients (91 %) in this cohort warranted a blood transfusion. In haemodynamically unstable patients a Pelvic binder or external fixation can help to limit the Pelvic bleeding, provide pain relief and improve nurseability, which is why the high rate of external fixation as initial treatment in this cohort is not surprising. However, if a patient is stable on admission, optimal anatomic reduction and Pelvic symmetry should be the goal of the surgical treatment.

Biomechanical studies show that extreme forces are necessary to cause a fracture in the paediatric pelvis, so when it comes to Open fractures it is evident that there is a substantial transmission of force involved [27]. This significant force not only contributes to the fracture itself but also raises the likelihood of concomitant injuries. Furthermore, one should always be aware of the potential for so-called ‘plastic deformation’ in the paediatric pelvis. Due to the increased elasticity provided by the high amount of collagen in the paediatric pelvis, the bone can deform instead of breaking, leading to Pelvic asymmetry [28,29]. While the potential for bone remodelling used to be part of the reason why non-operative treatment was often advocated for in Children with Pelvic fractures, more recent studies show that radiographic asymmetries do not correct over time [1]. This study shows similar results with high rates of growth disturbances, malunion, and ankyloses of the Pelvic joints that do not seem to improve over time. However, to which degree the radiographic asymmetries translate to musculoskeletal dysfunction remains unclear due to the small sample size. While there are higher SMFA dysfunction scores in patients who have radiographic malunion and asymmetry, some patients without many radiographic abnormalities have high dysfunction scores as well, which can probably be contributed to their concomitant injuries.

Furthermore, while the initial burden of the dysfunctions might seem minor due to the resilience of these Children, the results of the SMFA subdomain on problems in daily activities shows a varying level of impairment later in life. It should be noted that during the follow-up visits a discrepancy was seen in the self-reported complaints of the Children and adults, and their proxy. The proxy often reported more severe limitations in day to day life than the patient, which is a known problem in health-related quality of life instruments [30,31]. The clinical significance of this disparity in the context of Open paediatric Pelvic fractures remains unclear, but the current dysfunction scores might be an underestimation of the real burden these patients experience.

The mental and emotional problems seen in this cohort of patients are on par with the existing literature. Other studies have shown that 56 % of Children will be diagnosed with a psychiatric disorder after sustaining a Pelvic fracture, which can be attributed to the traumatic event that occurred as well as having to cope with lifelong disabilities [26].

The strength of this study is its focus on a relatively rare and complex condition, which adds valuable insights to the existing literature despite its small cohort size. The study offers a comprehensive assessment of both short-term clinical treatment and long-term functional outcomes and is, to the best of our knowledge, the first study that describes the long-term musculoskeletal function, neurological, and urogenital function outcomes of Children who suffered an Open Pelvic fracture. However, several limitations should be acknowledged. Firstly, the small sample size with only 11 patients included limits the generalizability of the results and may introduce selection bias. In addition, the retrospective nature of the study may introduce information bias if certain data points were not consistently documented in patient records. Furthermore, the follow-up period, which ranges from 4 to 191 months, lacks uniformity which might affect the accuracy of long-term outcome assessments. The low participation rate (11 out of 17 eligible patients) may introduce non-response bias, as those who declined to participate may have had different experiences or outcomes. Further research with larger cohorts is warranted to refine our understanding of these challenging cases and improve their management.

### Summary and conclusions

The results of this study show that Children with an Open Pelvic fracture are mostly female adolescents who were involved in a traffic accident with a large vehicle. The soft-tissue injuries involved the anorectum and vagina in most patients, and almost all patients (*n* = 10, 91 %) needed surgical intervention to repair these injuries. External fixation is a popular method to stabilize paediatric patients with an Open Pelvic fracture in the acute setting, however, direct Open reduction and internal fixation has become more preferred the last couple of years as a treatment method. Although these patients have severe soft-tissue injuries in the anogenital tract and often need multiple re-operations, their long-term urogenital function is mostly intact. All patients report some long-term musculoskeletal dysfunctions, which are most noticeable in the lower extremities and during daily activities.

## Ethical considerations

The study was approved by the Medical Research Ethics Committee Erasmus MC (ref. No MEC-2021-0570) and Board of Directors of Amsterdam UMC (ref. No. 2021_243) and has been conducted in accordance with to the principles of the Declaration of Helsinki accordance and the Medical Research Involving Human Subjects Act. Written informed consent was obtained from all included patients. If the patient was younger than 16 years at follow-up, parents or legal guardians also signed informed consent.

## CRediT authorship contribution statement

**A.H.M. Mennen:** Writing – original draft, Visualization, Project administration, Investigation, Formal analysis, Data curation. **E.M.M. Van Lieshout:** Writing – review & editing, Supervision, Project administration, Methodology, Formal analysis, Conceptualization. **P.A. Bisoen:** Data curation. **F.W. Bloemers:** Writing – review & editing. **A.E. Geerlings:** Data curation. **D. Koole:** Conceptualization. **M.H.J. Verhofstad:** Writing – review & editing. **J.J. Visser:** Writing – review & editing. **D. van Embden:** Writing – review & editing, Supervision, Conceptualization. **M.G. van Vledder:** Writing – review & editing, Supervision, Investigation, Conceptualization.

## Declaration of competing interest

The authors declare that they have no known competing financial interests or personal relationships that could have appeared to influence the work reported in this paper.
